# Release of HMGB1 from human-derived cancer and normal cells by internal targeted radiotherapy with ^131^I-*meta*-iodobenzylguanidine

**DOI:** 10.1093/jrr/rraf034

**Published:** 2025-06-30

**Authors:** Kakeru Sato, Ririka Handa, Jianwei Yao, Yuka Hirayama, Yuna Hamada, Jundai Yamagata, Taiga Watanabe, Asuka Mizutani, Hiroshi Wakabayashi, Masato Kobayashi, Ryuichi Nishii, Keiichi Kawai

**Affiliations:** Division of Health Sciences, Graduate School of Medical Sciences, Kanazawa University, 5- 11-80 Kodatsuno, Kanazawa 920-0942, Japan; Radiological Center, University of Fukui Hospital, 23-3 Matsuokashimoaizuki, Eiheiji, Fukui 910-1193, Japan; Division of Health Sciences, Graduate School of Medical Sciences, Kanazawa University, 5- 11-80 Kodatsuno, Kanazawa 920-0942, Japan; Division of Health Sciences, Graduate School of Medical Sciences, Kanazawa University, 5- 11-80 Kodatsuno, Kanazawa 920-0942, Japan; Division of Health Sciences, Graduate School of Medical Sciences, Kanazawa University, 5- 11-80 Kodatsuno, Kanazawa 920-0942, Japan; Division of Health Sciences, Graduate School of Medical Sciences, Kanazawa University, 5- 11-80 Kodatsuno, Kanazawa 920-0942, Japan; Division of Health Sciences, Graduate School of Medical Sciences, Kanazawa University, 5- 11-80 Kodatsuno, Kanazawa 920-0942, Japan; Division of Health Sciences, Graduate School of Medical Sciences, Kanazawa University, 5- 11-80 Kodatsuno, Kanazawa 920-0942, Japan; Faculty of Health Sciences, Institute of Medical, Pharmaceutical and Health Sciences, Kanazawa University, 5-11-80 Kodatsuno, Kanazawa 920-0942, Japan; Department of Nuclear Medicine, Kanazawa University Hospital, Kanazawa University, Takara-machi 13-1, Kanazawa, Ishikawa, 920-8641, Japan; Faculty of Health Sciences, Institute of Medical, Pharmaceutical and Health Sciences, Kanazawa University, 5-11-80 Kodatsuno, Kanazawa 920-0942, Japan; Department of Integrated Health Sciences, Graduate School of Medicine, Nagoya University, 1-1-20 Daiko Minami, Higashi-ku, Nagoya 461-8673, Japan; Faculty of Health Sciences, Institute of Medical, Pharmaceutical and Health Sciences, Kanazawa University, 5-11-80 Kodatsuno, Kanazawa 920-0942, Japan; Biomedical Imaging Research Center, University of Fukui, 23-3 Matsuokashimoaizuki, Eiheiji, Fukui 910-1193, Japan

**Keywords:** damage-associated molecular patterns, high mobility group box-1 protein, X-ray, ^131^i-*m*-iodobenzylguanidine, lactate dehydrogenase, immunotherapy

## Abstract

The rare abscopal effect in radiotherapy is thought to result from immune-activating damage-associated molecular patterns, such as high mobility group box-1 protein (HMGB1), released from cancer cells. While external irradiation of cancer cells increases HMGB1 release, it remains unclear whether internal radiotherapy with ^131^I-*meta*-iodobenzylguanidine (^131^I-MIBG) induces similar effects. This study aimed to determine if HMGB1 is released from human-derived cancer and normal cells after ^131^I-MIBG administration. The number of cells, extracellular lactate dehydrogenase (LDH) and HMGB1 were measured in H441 and human keratinocyte cell line (HaCaT) at 1 day after 2- and 10-Gy X-ray irradiation. Accumulations of ^131^I-MIBG in SH-SY5Y and HaCaT were measured at 60 min after ^131^I-MIBG (0.37, 1.85 and 3.7 MBq/well) administration. The number of cells, extracellular LDH and HMGB1 were measured at 1 day after ^131^I-MIBG treatment. Results: The total number of cells decreased in both H441 and HaCaT at 1 day after 10-Gy X-ray irradiation. Extracellular LDH and HMGB1 from H441 after 10-Gy X-ray irradiation were significantly increased, while no increase was observed in HaCaT after 2- and 10-Gy X-ray irradiation. After 1.85 MBq (~4-Gy by converting of PHITS simulation) and 3.7 MBq ^131^I-MIBG (8-Gy) administrations, the total number of cells decreased in both SH-SY5Y and HaCaT at 1 day after ^131^I-MIBG administration. Extracellular LDH and HMGB1 were both significantly increased in SH-SY5Y, but only extracellular LDH was significantly increased in HaCaT. HMGB1 was released from neuroblastoma cells but not from normal cells after ^131^I-MIBG administration. A combination of ^131^I-MIBG and immunotherapy may be feasible.

## INTRODUCTION

Radiotherapy using X-rays and chemotherapy provided by anticancer agents are the primary cancer treatment modalities. The abscopal effect, a phenomenon in which non-irradiated tumors, such as metastases, shrink and/or disappear, rarely occurs in radiotherapy [1, 2]. Although the mechanism of the abscopal effect has not been fully elucidated, damage-associated molecular patterns (DAMPs) released from irradiated cancer cells are thought to be involved [3]. These DAMPs could be released by stressed cancer cells when they are damaged by radiation or anticancer agents [3, 4]. Some DAMPs activate immunity whereas others suppress it [5, 6]. Dendritic cells activated by immunostimulatory proteins phagocytose deceased cancer cells, recognize cancer antigens and activate cytotoxic T cells to attack cancer cells [7]. Hence, the dendritic cells released from dead normal cells recognize the normal cells from which they were released and may attack them. Additionally, the cytotoxic T-cell attacks on cancer cells that cause the abscopal effect are easily inhibited by combining the PD-1 receptor on the surface of immune cells with the PD-L1 ligands on the cell membranes of cancer cells [8]. Immune checkpoint inhibitors, such as nivolumab, pembrolizumab and durvalumab, have attracted attention because they inhibit these immune checkpoints (PD-1 or PD-L1), maintain immune activity by preventing PD-1 or PD-L1 binding, and restore antitumor effects [9, 10]. To enhance the abscopal effect, it is crucial to release more immunostimulatory proteins in DAMPs, activate more immune cells, and develop more effective immune checkpoint inhibitors.

DAMPs that activate immunity include adenosine triphosphate (ATP), calreticulin (CRT), and high mobility group box-1 protein (HMGB1) [11]. Extracellular ATP binds to the P2X7 purinoreceptor expressed on immune cell membranes and activates immune cells [12]. CRT exposed on cancer-cell membranes signals dendritic cells and others via CD91, contributing to immune activation [5]. Therefore, ATP and CRT primarily contribute to cancer immunity by binding to specific receptors. In contrast, HMGB1 has affinity for various receptors, such as Toll-like receptors (TLR) 2, TLR4 and the receptor for advanced glycation end-products and can activate immune cells through multiple pathways [13]. In addition, external irradiation of cancer has been shown to damage cells and increase the release of HMGB1 [14]. In radiotherapy for cancer, ideally only cancer cells should be irradiated, but even with certain techniques, such as intensity-modulated radiotherapy, it is difficult to achieve no damage to all tissue, and studies are underway to reduce toxicity to normal tissue [15, 16]. Although it has been shown that HMGB1 is released in irradiated skin cells in mice [17], it is not clear if HMGB1 is released from normal cells in humans.

In addition to external irradiation, radiopharmaceutical therapy has received attention in recent years [18]. In preclinical studies, radioimmunotherapy combining radiopharmaceutical therapy (^177^Lu-DOTATATE or ^223^RaCl_2_) with immunotherapy was conducted and shown to provide better cancer treatment than with ^177^Lu-DOTATATE or ^223^RaCl_2_ alone [19]. ^131^I-*meta*-iodobenzylguanidine (^131^I-MIBG), an internal radiopharmaceutical therapy agent, is frequently used to treat neuroblastoma and is effective against refractory or recurrent neuroblastomas [20, 21]. Nevertheless, there are side effects, such as diminished bone marrow function and limited therapeutic effectiveness in specific patient populations [20, 22]. In radioimmunotherapy using internal radiopharmaceutical therapy, the release of DAMPs, including HMGB1, from damaged cancer and normal cells has not been examined in comparison with that observed for radioimmunotherapy using external radiotherapy. The study aim was to determine if HMGB1 can be released from damaged human-derived cancer and normal cells after administration of ^131^I-MIBG. The release of HMGB1 from damaged cancer cells may lead to improved cancer treatment, but HMGB1 released from damaged normal cells may contribute to or cause side effects.

## MATERIALS AND METHODS

### Cancer cell lines

The human-derived lung adenocarcinoma cell line H441 (American Type Culture Collection, Manassas, VA, USA) and the human-derived neuroblastoma cell line SH-SY5Y were used as cancer cells in this study along with the human keratinocyte cell line (HaCaT) as normal cells. H441 was cultured in RPMI-1640 medium (RPMI, FUJIFILM Wako Chemical, Osaka, Japan), SH-SY5Y was cultured in Eagle’s minimum essential medium (EMEM, FUJIFILM Wako Chemicals), and Ham’s F-12 (Ham’s, FUJIFILM Wako Chemicals) and HaCaT were cultured in Dulbecco’s modified Eagle’s medium (DMEM, FUJIFILM Wako Chemicals). RPMI and DMEM were mixed with 10% fetal bovine serum (FBS), 1% penicillin, and 1% sodium pyruvate. EMEM was added to Ham’s mixed with 15% FBS, 1% penicillin, and 1% sodium pyruvate. All cells were cultured in an incubator at 37°C with 5% CO_2_.

### Administration and accumulation of ^131^I-MIBG in SH-SY5Y and HaCaT cells

SH-SY5Y and HaCaT were seeded into 12-well plates at 1.0 × 10^5^ cells/well, and 1 day later, the cells were pre-incubated in phosphate-buffered saline (PBS, pH 7.4) for ~5 min. After pre-incubation, the cells were incubated in ^131^I-MIBG (0.37, 1.85 and 3.7 MBq/well) for 60 min at 37°C and then the cells were washed twice with PBS (*n* = 4). The cells were lysed with 0.1 N NaOH, and a gamma counter (AccuFLEX γ7000, Hitachi Aloka Medical, Tokyo, Japan) was used to measure intracellular radioactivity. The results were expressed as percentage injected dose (%ID) / number of living cells determined by an automatic cell counter (LUNA FX7TM; Logo Biosystems, Gyeonggi-do, South Korea).

### Measurement the number of cells

H441 and HaCaT were seeded into 12-well plates at 1.0 × 10^5^ cells/well, and ~1 day later, the cells were irradiated with 2 and 10-Gy X-rays from X-ray irradiation equipment (MBR1520R-3; Hitachi, Tokyo, Japan). Additionally, SH-SY5Y and HaCaT were seeded into 12-well plates at 1.0 × 10^5^ cells/well, and ~1 day later, the cells were pre-incubated in PBS for ~5 min. After pre-incubation, the cells were incubated in ^131^I-MIBG (0.37, 1.85 and 3.7 MBq/well) for 60 min at 37°C and then the cells were washed twice with PBS, and replaced with new medium. The cells at 1 day after X-ray irradiation and ^131^I-MIBG administration were stained with acridine orange/propidium iodide and the number of living and dead cells were measured with an automatic cell counter (LUNA-FX7™, Logos Biosystems, Gyeonggi-do, South Korea).

### Lactate dehydrogenase assay

X-ray irradiation and ^131^I-MIBG treatment were performed as in section: Measurement the number of cells. The extracellular lactate dehydrogenase (LDH) released from the cells 1 day after X-irradiation and ^131^I-MIBG administration was measured using an LDH assay kit (Nacalai Tesque, Tokyo, Japan) according to the manufacturer’s protocol (*n* = 4). Briefly, the supernatant of the cultured cells was placed in a 96-well plate, reacted with the reagent supplied with the kit, and the absorbance was measured using an absorbance microplate reader (SH-1300Lab, Hitachi High-Tech).

### HMGB1 assay

X-ray irradiation and ^131^I-MIBG treatment were performed as in section: Measurement the number of cells. The extracellular HMGB1 released from the cells 1 day after X-irradiation and ^131^I-MIBG administration was measured using an HMGB1 ELISA Kit Exp (Shino-Test, Tokyo, Japan) according to the manufacturer’s protocol (*n* = 4). Briefly, the supernatant of the cultured cells was placed in a 96-well plate, reacted with the reagent supplied with the kit, and the absorbance was measured using an absorbance microplate reader.

### Statistical analysis

GraphPad Prism 8 statistical software (GraphPad Software, Inc., La Jolla, CA, USA) was used to perform all statistical analyses. A two-tailed paired Student’s *t*-test was used to for comparisons between the two groups. Values of *P* ≤ 0.01 or ≤ 0.05 were accepted as indicating statistical significance.

## RESULT

**Fig. 1:**
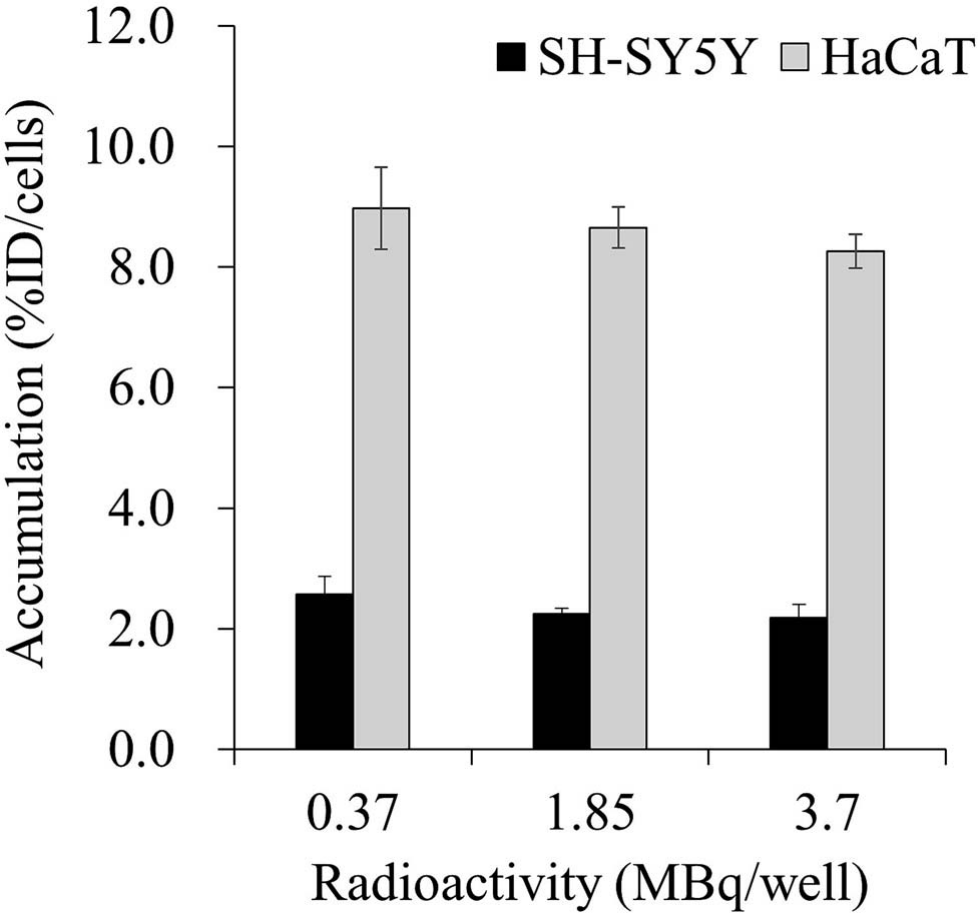
Accumulation of ^131^I-*meta*-iodobenzylguanidine (^131^I-MIBG) in SH-SY5Y and HaCaT at 60 min after administration. The accumulation of ^131^I-MIBG was constant regardless of the radioactivity administered. The accumulation of ^131^I-MIBG was greater in HaCaT than in SH-SY5Y.

Figure 1 shows the accumulation of ^131^I-MIBG in the SH-SY5Y and HaCaT cells at 60 min after administration. ^131^I-MIBG accumulated more in the HaCaT cells than in the SH-SY5Y cells, and the accumulation of ^131^I-MIBG in both the SH-SY5Y and HaCaT cells was not significantly affected by the radioactivity of the administered ^131^I-MIBG.

Table 1 shows the number of cells after X-ray irradiation. The number of total and living cells decreased at 1 day after 10-Gy X-ray irradiation for both H441 and HaCaT. The number of dead cells in H441 increased at 1 day after 10-Gy X-ray irradiation, but no significant change was observed in HaCaT. There was no change in either H441 or HaCaT at 1 day after 2-Gy X-ray irradiation. Table 2 shows the number of cells after ^131^I-MIBG administration. There were no significant changes in the number of cells in SH-SY5Y and HaCaT at 1 day after ^131^I-MIBG administration of 0.37 MBq. On the other hand, total, living, and dead cells were significantly decreased in SH-SY5Y and HaCaT at 1 day after ^131^I-MIBG administration of 1.85 and 3.7 MBq.

**Table 1:** The number of cells in H441 and HaCaT at 1 day after X-ray irradiation

		The number of cells (×10^5^)		
		Control	2-Gy	10-Gy
H441	Total cells	63.8 ± 9.2	59.4 ± 8.8	27.9 ± 3.3^**^
	Living cells	57.4 ± 8.2	54.5 ± 8.1	19.2 ± 2.4^**^
	Dead cells	6.4 ± 1.1	4.9 ± 0.9	8.7 ± 1.3^*^
HaCaT	Total cells	40.2 ± 2.4	39.3 ± 1.9	18.2 ± 0.5^**^
	Living cells	38.0 ± 2.7	36.5 ± 2.5	16.6 ± 1.1^**^
	Dead cells	2.2 ± 0.4	2.8 ± 0.6	1.5 ± 0.7

The number of total and living cells decreased after 10-Gy X-ray irradiation for both H441 and HaCaT. The number of dead cells in H441 increased, but no significant change was observed in HaCaT. In addition, there was no change in either H441 or HaCaT after 2-Gy X-ray irradiation. ^*^P < 0.05 and ^**^P < 0.01 versus the controls.

**Table 2:** The number of cells in SH-SY5Y and HaCaT at 1 day after ^131^I-MIBG administration

		The number of cells (×10^5^)			
		Control	0.37 MBq	1.85 MBq	3.7 MBq
SH-SY5Y	Total cells	49.4 ± 11.1	47.0 ± 9.1	33.7 ± 5.2^*^	27.3 ± 2.7^*^
	Living cells	47.4 ± 11.3	44.4 ± 9.7	32.8 ± 4.9^*^	26.6 ± 2.6^*^
	Dead cells	2.0 ± 0.6	2.6 ± 2.2	0.9 ± 0.5^*^	0.7 ± 0.1^*^
HaCaT	Total cells	24.3 ± 8.3	25.6 ± 3.7	16.7 ± 2.5^**^	11.4 ± 1.4^**^
	Living cells	22.9 ± 1.5	24.5 ± 3.2	16.2 ± 2.6^**^	11.0 ± 1.3^**^
	Dead cells	1.4 ± 0.3	1.2 ± 0.8	0.5 ± 0.2^**^	0.3 ± 0.3^**^

There were no significant changes in the number of cells in SH-SY5Y and HaCaT after ^131^I-MIBG administration of 0.37 MBq. The number of total, living, and dead cells were significantly decreased in SH-SY5Y and HaCaT after ^131^I-MIBG administration of 1.85 MBq and 3.7 MBq. ^*^P < 0.05 and ^**^P < 0.01versus the controls.

**Fig. 2:**
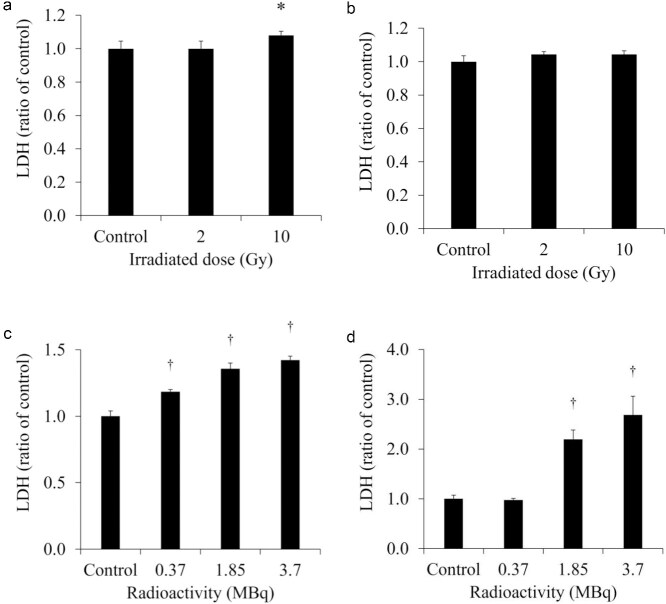
The extracellular lactate dehydrogenase (LDH) released in the culture medium of H441 (a) and HaCaT (b) at 1 day after X-ray irradiation and of SH-SY5Y (c) and HaCaT (d) at 1 day after ^131^I-MIBG administration. The LDH released was increased in H441 treated with 10-Gy X-ray irradiation but HaCaT was unchanged for both 2-Gy and 10-Gy irradiation. LDH released from SH-SY5Y was significantly increased after administration of 0.37 MBq ^131^I-MIBG, and the increase was greater with higher radioactivity. LDH released from HaCaT was not increased after administration of 0.37 MBq ^131^I-MIBG but was predominantly increased at 1.85 and 3.7 MBq ^131^I-MIBG. ^†^*P* < 0.01 and ^*^P < 0.05 versus the controls.

**Fig. 3:**
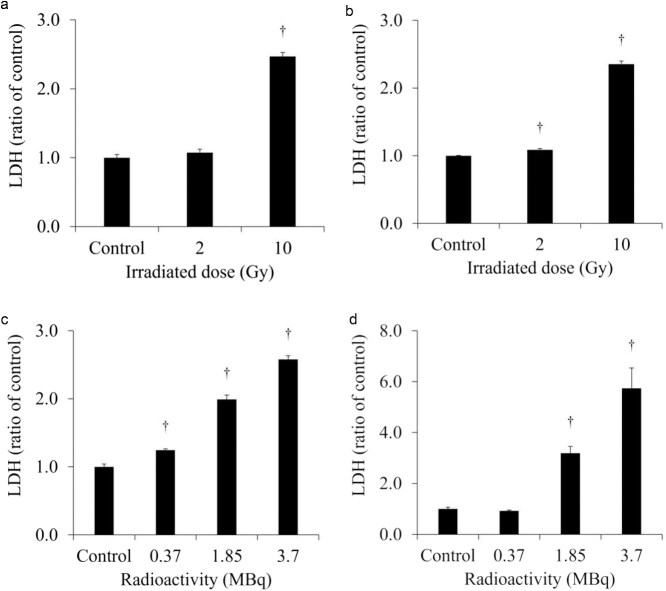
The extracellular LDH released per cell, normalized for the number of total cells. The LDH released per cell was increased H441 (a) and HaCaT (b) at 1 day after 10-Gy X-ray irradiation. The LDH released per cell was significantly increased after 2-Gy X-ray irradiation in HaCaT. In addition, the LDH released per cell increased at all examined ^131^I-MIBG radioactivity’s in SH-SY5Y (c), and the LDH released per cell increased at 1.85 and 3.7 MBq of ^131^I-MIBG in HaCaT (d).

The extracellular LDH released in the culture medium of each cell is shown in Fig. 2. The extracellular LDH released from H441 was increased at 1 day after 10-Gy X-ray irradiation. However, the extracellular LDH released from HaCaT was not increased at 1 day after 2- and 10-Gy X-ray irradiation. The extracellular LDH released from SH-SY5Y was significantly increased after administration of 0.37 MBq ^131^I-MIBG and the increase was greater with the higher radioactivity. On the other hand, the extracellular LDH released from HaCaT was not increased after administration of 0.37 MBq ^131^I-MIBG but was predominantly increased at 1.85 and 3.7 MBq ^131^I-MIBG. The degree of increase in the release of LDH was much greater in HaCaT than in SH-SY5Y. Figure 3 shows the extracellular LDH released per cell, normalized for the number of total cells. The extracellular LDH released per cell was increased not only from H441 but also from HaCaT at 1 day after 10-Gy X-ray irradiation. In addition, the extracellular LDH released per cell from HaCaT was significantly increased even after 2-Gy X-ray irradiation. The extracellular LDH released per cell increased at all examined ^131^I-MIBG radioactivity’s in SH-SY5Y, and the extracellular LDH released per cell increased at 1.85 and 3.7 MBq of ^131^I-MIBG in HaCaT cells.

**Fig. 4:**
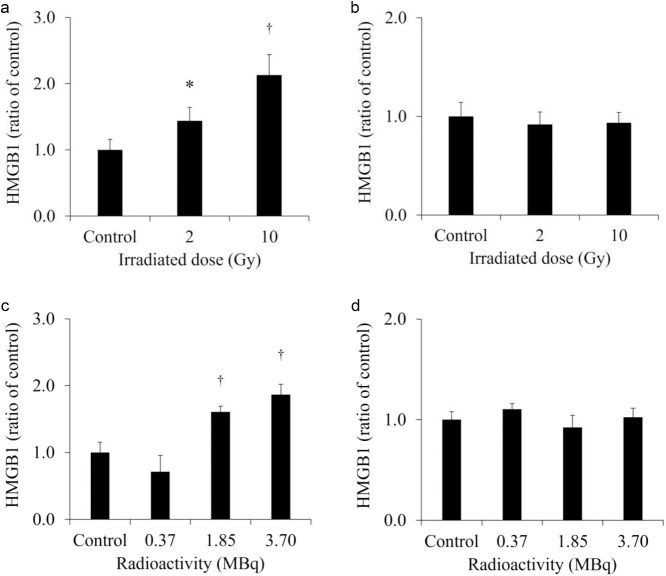
The extracellular HMGB1 in the cultured medium of H441 (a) and HaCaT (b) at 1 day after X-ray irradiation, and SH-SY5Y (c) and HaCaT (d) at 1 day after ^131^I-*m*-iodobenzylguanidine (^131^I-MIBG) administration. HMGB1 release was significantly increased in H441 irradiated with 2-Gy and 10-Gy X-ray irradiation, but HaCaT was unchanged for both 2-Gy and 10-Gy. HMGB1 release was significantly increased after administering 1.85 MBq and 3.7 MBq of ^131^I-MIBG, but was unchanged after all radioactivity. ^†^*P* < 0.01 and ^*^P < 0.05 versus the controls.

**Fig. 5:**
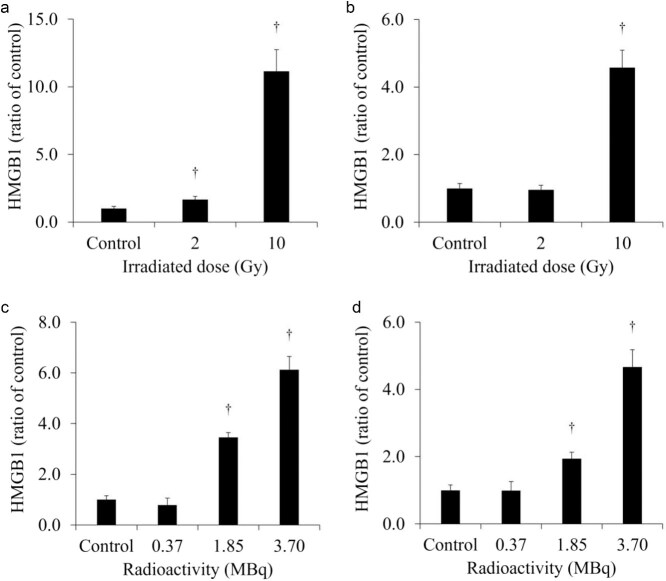
The extracellular HMGB1 released per cell, normalized for the number of total cells. The HMGB1 released per cell was increased in H441 (a) and HaCaT (b) at 1 day after 10-Gy X-ray irradiation. The HMGB1 released per cell was significantly increased after 2-Gy X-ray irradiation. In addition, the HMGB1 released per cell increased at 1.85 and 3.7 MBq of ^131^I-MIBG in SH-SY5Y (c) and HaCaT (d).

Figure 4 shows the extracellular HMGB1 in the culture medium of each cell line. The release of HMGB1 from H441 was significantly increased for both 2- and 10-Gy X-ray irradiation. Moreover, the release of HMGB1 from H441 was higher after 10-Gy than after 2-Gy X-ray irradiation. In contrast, there was no change in HMGB1 release in HaCaT after X-ray irradiation. Although there was no change in the HMGB1 release from SH-SY5Y administered 0.37 MBq of ^131^I-MIBG, HMGB1 release was significantly increased with 1.85 and 3.7 MBq of ^131^I-MIBG. However, there was no change in HMGB1 release in HaCaT administered ^131^I-MIBG at any radioactivity dose. Figure 5 shows the extracellular HMGB1 released per cell, normalized for the number of total cells. The extracellular HMGB1 released per cell was increased not only from H441 but also from HaCaT at 1 day after 10-Gy X-ray irradiation. In addition, the extracellular HMGB1 released per cell from H441 was significantly increased even after 2-Gy X-ray irradiation. ^131^I-MIBG administration of 1.85 and 3.7 MBq increased the extracellular HMGB1 released per cell in SH-SY5Y and HaCaT.

## DISCUSSION

Although the use of radioimmunotherapy, which combines external radiotherapy with immunotherapy, is a known treatment option, only a few studies have combined immunotherapy with radiopharmaceutical therapy. Immunotherapy is initiated by the release of immunostimulatory DAMPs from cancer cells, but it has not been known if radiopharmaceutical therapy causes the release of immunostimulatory DAMPs from cancer cells. This study aimed to determine if HMGB1 would be released from human-derived cancer cells and normal cells after ^131^I-MIBG administration, with the goal of establishing useful radioimmunotherapy in the form of immunotherapy combined with ^131^I-MIBG, which has rarely been studied. Therefore, H441, a human-derived lung adenocarcinoma cell line that is an indication for radiotherapy [23], and SH-SY5Y, a human-derived neuroblastoma cell line that is an indication for radiopharmaceutical therapy using ^131^I-MIBG, were selected on the basis of previous research [18, 20] as the cancer cell lines in the present study. HaCaT cells established from adult male skin were used as the normal cell line because normal cells are usually difficult to culture [24]. The HaCaT cells have expressions of organic cation transporters (OCTs) [25], which is transport mechanism of ^131^I-MIBG, at the cell membrane. In addition, X-ray irradiation for cancer treatment provides an influence on the skin.

Assuming conventional external radiotherapy, we selected a 2-Gy X-ray dose, which is often used for single-dose conventional therapy. Additionally, assuming stereotactic body radiotherapy (SBRT) with a higher single-dose of radiation, we selected 10-Gy as the X-ray dose, which is commonly used in SBRT [26, 27]. SBRT is frequently used to treat lung cancer [28]. In the present study, we observed that the extracellular LDH released from H441 was increased at 1 day after 10-Gy X-ray irradiation (Fig. 2). However, the extracellular LDH released from HaCaT was not increased at 1 day after 2- and 10-Gy X-ray irradiation. LDH is an enzyme that catalyzes conversion of pyruvate to lactate in the glycolytic process [29]. Normally, LDH remains in the cytoplasm, but when the cell membrane is damaged, LDH is excreted out of the cell [30]. Hence, measurement of LDH release is a good method for detecting cell death. In addition, the number of total and living cells was decreased in both H441 and HaCaT at 1 day after 10-Gy X-ray irradiation (Table 1). This suggests that the cells were stressed by X-ray irradiation and the cell cycle was arrested. The extracellular HMGB1 release from H441 was significantly increased at 1 day after 2- and 10-Gy X-ray irradiation (Fig. 4). Moreover, the increase was more pronounced for 10-Gy than for 2-Gy irradiation. However, the amount of HMGB1 released from the HaCaT cells by irradiation did not change for both 2-Gy and 10-Gy X-ray irradiation. These results suggest that 10-Gy X-ray irradiation disrupted the cell membranes of H441 cells and released HMGB1. The molecular weight of LDH is 140 kDa, while that of HMGB1 is 30 kDa [31, 32]. Hence, it is possible that at low doses such as 2-Gy, the cell membranes were less damaged and the low-molecular-weight HMGB1 could pass but not LDH. HaCaT cell death was not observed at 1 day after X-ray irradiation, and HMGB1 release did not occur. The extracellular LDH and HMGB1 per cell, normalized for number of total cells, increased after 10-Gy X-ray irradiation for both H441 and HaCaT. The cells that were damaged and released LDH and HMGB1 during the 1 day after X-ray irradiation but subsequently recovered were counted as living cells, so the number of total cells was normalized. This suggests that HaCaT also has the ability to release HMGB1. However, while the extracellular HMGB1 per cell increased, the total extracellular HMGB1 is the critical factor, as immune cells are only activated when the overall amount of HMGB1 changes. In actual clinical radiotherapy, irradiating lung cancer with 10-Gy (assuming SBRT) does not necessarily mean that the skin is exposed to 10-Gy of X-ray irradiation. Immune cells activated by DAMPs, such as HMGB1, recognize the cells from which it was released and attack living cells. Therefore, by maximizing the HMGB1 released only from cancer cells, it could be possible to suppress damage to the skin and inflict further damage only to the tumor.

In the present study, we observed that the radiopharmaceutical therapeutic agent ^131^I-MIBG had accumulated more in HaCaT cells than in SH-SY5Y cells at 60 min after administration (Fig. 1). ^131^I-MIBG is mainly taken up into cells via the norepinephrine transporter, which has been shown to be abundantly expressed in neuroblastoma cells [33]. In addition, ^131^I-MIBG has an affinity for the influx drug transporter organic cation / carnitine transporter (OCTN)1, OCTN2, OCT1–3, and the efflux drug transporter multidrug resistance proteins (MRP) 1 and MRP4 [34, 35]. Therefore, the high accumulation of ^131^I-MIBG in HaCaT cells may be due to the high expression of OCTNs and OCTs. It is also possible that the expression of MRPs is higher in SH-SY5Y than in HaCaT cells. The extracellular HMGB1 released from SH-SY5Y was significantly increased after administration of 1.85 and 3.7 MBq ^131^I-MIBG. Shinohara *et al*. showed that treatment with 3.0 MBq of ^131^I-MIBG for 3 days reduced cell viability by ~50% *in vitro* study [36]. In this study, we selected 0.37 MBq, 1.85 MBq, which is smaller radioactivity than 3.0 MBq, and 3.7 MBq, which is similar radioactivity with 3.0 MBq, to examine HMGB1 release from SH-SY5Y. Similarly, the extracellular LDH was significantly increased after administration of all ^131^I-MIBG radioactivates. Furthermore, the number of total, living and dead cells were decreased in SH-SY5Y at 1 day after administration of 1.85 MBq and 3.7 MBq ^131^I-MIBG (Table 2). These results suggest that ^131^I-MIBG administration caused cell death and the release of HMGB1. However, In SH-SY5Y, 0.37 MBq of ^131^I-MIBG significantly reduced LDH but hardly released HMGB1. Given that LDH is localized in the cytoplasm and HMGB1 in the nucleus [30, 37], LDH is readily released into the extracellular space upon cell membrane damage, whereas HMGB1 release requires a two-step process involving nuclear-to-cytoplasmic translocation followed by cell membrane disruption. It has been established that under cellular stress, HMGB1 undergoes acetylation, interacts with Exportin 1 (XPO1), and is actively transported from the nucleus to the cytoplasm [38]. Furthermore, Shinohara *et al*. calculated the absorbed dose of ^131^I-MIBG to the cells using the PHITS simulation, which was 12.4 x 10^−12^ Gy/Bq/ml per β-ray emitted from ^131^I-MIBG [36]. In this study, 3.7 MBq/500 μl was administered to the cells, resulting in an absorbed dose of ~92 × 10^−6^ Gy per s, and a total absorbed dose of ~8-Gy from the ^131^I-MIBG was taken up for 1 day. It is likely that 0.37 MBq will be an absorbed dose of ~0.8-Gy. Accordingly, such low doses may be insufficient to induce HMGB1 acetylation, resulting in its retention within the nucleus. Nonetheless, minor plasma membrane damage was observed even at these low doses, which may account for the observed LDH release.

In HaCaT, the extracellular LDH was also significantly increased after administration of 1.85 and 3.7 MBq ^131^I-MIBG, and the degree of increase in the release of LDH was much greater in HaCaT than in SH-SY5Y. The accumulation of ^131^I-MIBG also was greater in HaCaT than in SH-SY5Y, suggesting that more cell death and LDH release occurred in HaCaT. However, no HMGB1 was released from HaCaT after the administration of ^131^I-MIBG. Park *et al*. showed that HMGB1 was significantly released from HaCaT 24 hours after ultraviolet irradiation and that hemin loading suppressed HMGB1 release [39]. Hence, HaCaT does not necessarily release HMGB1. In addition, the extracellular HMGB1 per cell, normalized for the number of total cells, increased after administration of 1.85 and 3.7 MBq ^131^I-MIBG in HaCaT (Fig. 5). This suggests that HaCaT also has the ability to release HMGB1 by β-ray irradiation. We mentioned as above, while the extracellular HMGB1 per cell increased, the total extracellular HMGB1 is the critical factor, as immune cells are only activated when the overall amount of HMGB1 changes. Furthermore, although this study focused on HMGB1, one of the DAMPs that activate immunity through various pathways, other DMAPs, such as ATP and CRT, possible were released in increased amounts in HaCaT.

It was shown that reducing the radioactivity of administered ^131^I-MIBG may help reduce side effects by minimizing damage to normal cells caused by attacking cancer cells, not only because of the damage caused by β-ray radiation, but also because of the damage caused by activation of immune cells induced by HMGB1 release. However, elucidation of the mechanism by which HMGB1 release is caused by X-ray irradiation and the radiopharmaceutical therapeutic agent dose is not clear and requires further investigation. In addition, it would ultimately be desirable to conduct *in vivo* studies as well as *in vitro*. *In-vivo* studies, HMGB1 released from cells by X-ray irradiation and ^131^I-MIBG administration is released into the blood. However, there is no method to distinguish whether the released HMGB1 is from tumor-derived or normal cell-derived HMGB1, and the exact amount of HMGB1 released cannot be measured *in-vivo* studies. Therefore, our *in vitro* experiments were conducted to determine the amount of increased HMGB1 released from only cancer cells and normal cells. In addition, HaCaT was used in this study due to the difficulty of culturing normal cells, but considering the possibility of side effects such as myelosuppression and gastrointestinal symptoms after ^131^I-MIBG administration, the use of normal bone marrow cells and intestinal cells should be investigated in the future.

Another limitation is that a simple comparison of X-rays and β-rays is not possible. We selected 2-Gy, which is often used for single-dose conventional therapy, and 10-Gy, which is assumed for stereotactic radiotherapy assuming external X-ray irradiation. For the internal radiotherapy with ^131^I-MIBG, 0.37, 1.85 and 3.7 MBq were selected based on the study by Shinohara *et al* [36]. Based on our results, we can estimate that the absorbed dose is ~8-Gy. The absorbed dose from a dose of 3.7 MBq ^131^I-MIBG would be similar with 10-Gy X-ray irradiation. However, since it is assumed that the ^131^I-MIBG is also excreted out of the cells during the 1 day, the actual absorbed dose would be >8-Gy. In this experiment, a commonly X-ray irradiation equipment was used. The maximum tube voltage is 150 kV, and the maximum X-ray energy is 150 keV. On the other hand, β-ray from ^131^I-MIBG have an energy of 606 keV, four times higher than that of X-ray. In other words, the energy of X-ray irradiation and β-ray from ^131^I-MIBG is different. Based on these facts, we can say that a simple comparison between X-ray and β-ray is not possible. In the future, we would like to consider studies that allow us to compare the doses of external X-ray irradiation and nuclear medicine therapeutic agents from various perspectives.

In conclusion, HMGB1 was released in human-derived lung cancer cells after X-ray irradiation and in human-derived neuroblastoma cells after ^131^I-MIBG administration, but HMGB1 was not released in normal human epidermal keratinocyte cells under the same conditions. These findings indicate that the abscopal effect that can occur after X-ray irradiation and ^131^I-MIBG administration possibly enhances the cancer therapeutic effect without causing excessive damage to normal cells.

## Presentation at a conference

We presented these data in the 12th China-Japan-Korea Symposium on Radiopharmaceutical Sciences (CJKSRS).
